# An Evaluation Method for Influential Nodes Based on Multi-Attribute Neighbor Contributions in Complex Networks

**DOI:** 10.3390/e28080935

**Published:** 2026-08-21

**Authors:** Na Zhao, Chao Dai, Guolin Yang, Ting Luo, Nifei Xiong, Jian Wang

**Affiliations:** 1 School of Software, Yunnan University, Kunming 650504, China; 2 College of Information Engineering and Automation, Kunming University of Science and Technology, Kunming 650500, China

**Keywords:** influential node identification, complex networks, local structural attributes, global structural attributes, multi-attribute fusion, neighbor contribution

## Abstract

Accurately identifying influential nodes is essential for analyzing network structures and optimizing information propagation. Existing methods predominantly rely on single indicators such as degree, H-index, or k-shell, inherently limiting their ability to capture a node’s true influence. Recent hybrid centrality approaches attempt to address this by combining multiple local and global attributes; however, they typically integrate features through simple weighting or superposition, failing to characterize the intrinsic synergy among structural properties. Furthermore, they often quantify neighbor contributions too coarsely, overlook the regulatory role of edge strength, and some suffer from high computational complexity, limiting scalability. To overcome these deficiencies, we propose WKDH, a novel influential node identification method based on multi-attribute neighbor contributions. WKDH fuses local structural attributes (degree and H-index) with global structural attributes (k-shell) via a multiplicative weighted synergy model, simultaneously capturing local connection “quantity,” local connection “quality,” and global core-layer position. By transforming neighbors’ comprehensive characteristics into regulated contribution degrees, WKDH mitigates excessive self-attribute interference and accurately reflects the actual propagation potential of edges. Notably, the method achieves linear computational complexity of O(m). Experimental results on nine real-world and six artificial networks demonstrate that WKDH outperforms nine established indicators in terms of node influence ranking, identification of high-influence nodes, and measuring propagation capability. Moreover, WKDH exhibits strong universality across diverse network structures, as it operates without parameter tuning.

## 1. Introduction

In the information age, complex networks widely exist in various fields such as social networks [1], transportation systems [2], and disease transmission [3]. Influential nodes within these systems act as hubs for information dissemination, sources of contagion, or vulnerabilities in structural robustness [4]. Identifying such nodes is therefore essential for analyzing network topology, optimizing propagation paths, preventing and controlling disease diffusion, and enhancing network anti-interference capability [5,6,7,8]. Node influence identification is typically quantified through centrality measures [9,10,11]. Existing centrality methods can generally be divided into four categories: local centrality [12], semi-local centrality, global centrality [13], and hybrid centrality [2,14,15].

Local centrality methods rely only on first-order neighborhood information of nodes. While computationally efficient, they fail to capture the global structure. For instance, degree centrality [16] considers only the number of connections, ignoring differences in neighbor quality and network position. H-index [17] incorporates neighbor degrees but remains restricted to local evaluation. The k-shell method [18] can distinguish the core layer positions of nodes but cannot differentiate the influence differences among nodes within the same shell layer. To address this limitation, researchers have proposed various more scientific methods for distinguishing node importance, such as weighted k-shell method based on potential edge weights (WKS) [19], weighted k-shell degree neighborhood method (WKSD) [20], and k-shell iteration factor [21], etc. [22,23]. These methods effectively distinguish nodes with the same k-shell value by introducing a weighting mechanism, thereby more accurately identifying influential nodes in complex networks.

Semi-local centrality methods extend based on local features, balancing locality and global perspective by incorporating multi-order neighborhood or local substructure information. For example, RASP evaluates node influence using the relative size of the average shortest path distance between a node and all other nodes in the network [24]; LRASP focuses on the relative change in the average shortest path within the local neighborhood after node removal to assess node influence [25,26]; and SAASP [27] uses an augmented graph to merge global semantic similarity and adopts a semi-local structure to effectively identify influential nodes. Other semi-local approaches introduce the idea of the gravity model to further enhance the feature integration of multi-order neighborhood information. These methods regard the local attributes of nodes as mass and the path length between nodes and their multi-order neighbors as spatial distance, quantifying the indirect association strength between nodes through the gravity formula. The final score of a node is obtained by accumulating the weighted gravity values of multi-order neighbors. Models such as IGC, GGC, and LHGC [28,29,30,31,32,33] refine the concepts of distance and mass in the gravity model from different perspectives. Semi-local centrality methods break through the limitation of first-order neighborhoods while avoiding the high complexity of global computation. However, their evaluation perspective is always limited by the preset local boundaries, and their effectiveness is highly dependent on network structure and scale, resulting in poor robustness and adaptability.

Global centrality methods take the overall network topological structure information as the core basis and evaluate the influence or importance of nodes by comprehensively considering their roles and positions in the overall connection pattern of the network. These methods are not limited to the local area around the node but regard the entire network as an organic whole, exploring the key value of nodes from a global perspective. For example, classic global centrality methods such as closeness centrality and betweenness centrality [34,35,36,37]. Closeness centrality uses the average shortest path distance from a node to all other nodes in the network as the evaluation index. Betweenness centrality measures the importance of a node as a bridge connecting different parts of the network by counting the frequency of the node appearing in all shortest paths of the network. These methods capture nodes’ overall structural significance, offering valuable insights into global network function. However, global centrality methods also have obvious limitations. The most prominent problem is their extremely high computational complexity; most global centrality methods have a computational complexity of *O*(*n*^^2^^) or even higher, making them difficult to apply in large-scale networks. To reduce computational complexity, drawing on the recursive idea of eigenvector centrality, researchers have proposed algorithms such as PageRank [38] and TrustPR [39], etc. [40,41], which have significant effects in directed networks. However, these algorithms perform poorly in undirected networks [2].

Centrality indices have specific application scenarios [42], and there is no single index that can be universally applied to all network structures. The diversity of node embeddings within a network necessitates measuring different node characteristics with different centrality indices. Since individual centrality indices cannot capture a wide range of node characteristics, designing a hybrid centrality method that includes multiple individual indices has been proven to be an effective way to enhance the effect of node influence identification [43,44,45]. Therefore, indices with low computational cost and effective reflection of key node characteristics are favored in hybrid centrality methods. Indices such as DC [16], H-index [17], and k-shell [18,46,47] are often used in hybrid centrality methods due to their excellent scalability. For example, GSM [48] uses the natural exponential of the node’s *k*-Shell index normalized by the network size as self-influence $SI(v_{i})$, and the aggregated *k*-Shell values of all other nodes weighted by the reciprocal shortest path distance as global influence $GI(v_{i})$. It then obtains the final score through the product of self-influence and global influence, $GSM\left(v_{i}\right)=SI\left(v_{i}\right)\times GI\left(v_{i}\right)$. WDKS [49] defines intrinsic influence as the product of node degree and k-shell, and incorporates the centrality influence of neighbor nodes through potential edge weights, combining the two to evaluate node influence. Similarly, methods such as EHC [50] and LCH [51,52] leverage combinations of multiple single feature indices to enhance the ability to identify node influence without significantly increasing computational complexity, indicating that hybrid centrality methods are an effective means to solve node identification problems.

In parallel with the above topological hybrid approaches, the past few years have witnessed a surge of interest in applying machine learning (ML) and deep learning to influential node identification. Stolarski et al. [53] showed that ML classifiers trained on topological descriptors can surpass classical centrality measures in predicting influence spread, suggesting that data-driven strategies constitute a promising frontier. Graph neural networks and network embedding techniques further enable end-to-end learning of node importance from complex structural patterns. Despite their predictive power, these methods generally demand abundant labeled training data, costly iterative optimization, and high computational resources, which restricts their scalability in large-scale or dynamically evolving networks. In addition, the lack of explicit structural interpretability in “black-box” ML models complicates the extraction of actionable topological insights. Consequently, there remains a pressing need for lightweight, interpretable, and parameter-free topological methods that can deliver competitive accuracy without training overhead.

However, existing hybrid centrality methods still face limitations, which persist even in recent advances. For instance, WDKS [49] combines degree and k-shell via simple product and sums weighted neighbor contributions, yet its edge weights ignore neighbor quality (e.g., H-index) and lack a synergy mechanism among the three structural dimensions. HNPR [54] integrates k-shell with PageRank, but its multi-layer multiplication introduces score instability and remains ineffective in undirected networks. HIC [55] and LSS [56] incorporate richer local features (clustering coefficient or triangle counts), yet they rely on direct summation of heterogeneous attributes without modeling their intrinsic interactions; moreover, LSS incurs semi-local complexity $O(n{\langle k\rangle }^{2}+|E|)$. EWKSD [57] introduces a balance factor λ, but this parameter requires empirical tuning across different network densities. Consequently, a fundamental gap remains: no existing method simultaneously (i) captures the multiplicative synergy among local quantity (degree), local quality (H-index), and global position (k-shell); (ii) quantifies neighbor contributions through edge-strength regulation rather than self-attribute reuse; and (iii) achieves linear O(m) complexity without parameter tuning. To address the defects of the existing methods, this paper proposes an influential node evaluation method based on multi-attribute neighbor contributions, aiming to balance identification accuracy and computational efficiency. This method innovatively integrates the local structural attributes and global structural attributes of nodes to construct a weighted evaluation model. Node “quality” and “quantity” are captured through the product of the node’s H-index and degree, and combines the k-shell value to reflect the global core layer position. These attributes jointly define the potential weight of edges. It characterizes the synergistic effect through the product relationship between attributes, avoiding the limitation of simple superposition; converts the comprehensive characteristics of neighbor nodes into the contribution degree to the target node, takes neighbor contribution as the core evaluation index, avoids excessive interference from the node’s own attributes, and solves the problem of insufficient computational efficiency in large-scale networks through linear traversal of edges and neighbors, making it applicable to large-scale networks. The specific contributions of this paper are as follows:

1. A multiplicative synergy indicator (KDH) that fuses degree, H-index, and k-shell to capture the intrinsic interaction among local “quantity,” local “quality,” and global core-layer position, overcoming the limitation of linear weighting or superposition in existing hybrid methods.
2. An edge-strength-regulated neighbor contribution mechanism that converts neighbors’ comprehensive characteristics into normalized contribution degrees, mitigating self-attribute interference and accurately reflecting actual edge propagation potential.
3. Linear O(m) complexity with an adaptive, parameter-free fusion weight determined by average network topology, ensuring high scalability and robustness across diverse networks.
4. Data analysis on nine real-world and six artificial networks shows that WKDH outperforms nine existing indices in terms of node influence ranking, identification of highly influential nodes, and propagation capability.

The subsequent chapter structure of this paper is organized as follows: Section 2 introduces the baseline models used in the research; Section 3 details the rationale and core formulas of the WKDH method in detail; Section 4 presents the data analysis of real networks and artificial networks; and Section 5 summarizes the research.

## 2. Related Work

A central question in the field of complex network analysis is how to accurately and efficiently quantify node importance. Research on node influence evaluation has evolved from relying on single structural features to integrating multi-dimensional attributes. This section introduces the baseline centrality measures used in our study, reviews related progress, and analyzes the limitations of existing approaches.

### Baseline Centrality Measures

**Measure 1. Global importance of nodes (GIN).**

Conventional centrality measures focus merely on local or global topology separately, often overlooking nodes that are locally insignificant yet crucial for global connectivity. To address this, GIN [58] integrates self-importance and global influence, defined as $SIM_{i}=\exp\left(\alpha k_{i}/n\right)$ and ${\mathrm{GIM}}_{i}=\beta \sum_{j\neq i}k_{j}/d_{ij}$, respectively, where $d_{ij}$ denotes the shortest path distance between nodes *i* and *j*. The overall centrality is ${\mathrm{GIN}}_{i}={\mathrm{SIM}}_{i}\times {\mathrm{GIM}}_{i}$ for connected nodes and 0 otherwise. As in [58], α and β are set to 1.

**Measure 2. Weighted k-shell centrality (WKS).**

Due to each edge in k-shell centrality having a weight of 1, many nodes end up with the same k-shell value. To address this issue, WKS [19] further distinguishes nodes by calculating their weighted degrees based on potential edge weights. The potential edge weight is defined as $w_{ij}=k_{i}+k_{j}$, where $k_{i}$ and $k_{j}$ represent the degrees of nodes i and j. The weighted degree of a node is given by $kw_{i}=\lambda k_{i}+\left(1-\lambda \right)\sum_{j\in N_{i}}w_{ij}$. As referenced in [19], λ is set to 0.5.This weighting better differentiates nodes with identical k-shell values.

**Measure 3. Weighted neighborhood centrality (WNC).**

WNC [59] considers the diffusion importance of edges in the network. The diffusion importance of an edge is defined as $w_{ij}=k_{i}k_{j}$, where $k_{i}$ and $k_{j}$ are the k-shell values of nodes *i* and *j*. The influence of node *i* is calculated as $c\phi_{i}=\phi_{i}+\sum_{j\in N_{i}}\frac{w_{ij}}{\langle w\rangle }\phi_{j}$, where $\phi_{i}$ is the baseline centrality of node *i* (representing degree or k-shell in reference [59]; set as k-shell in this study); 〈w〉 is the average diffusion importance of all edges, meaning that the influence of neighbors is superimposed on the node’s intrinsic influence according to the diffusion importance of edges.

**Measure 4. Weighted k-shell degree neighborhood centrality (WKSD).**

WKSD [20] combines a node’s degree, k-shell value, and the weighted sum of its first-order neighborhood to enhance sensitivity to local substructures in the network. The expression for the potential edge weight is $w_{ij}=\left(\alpha \cdot k_{i}+\mu \cdot ks_{i}\right)\cdot \left(\alpha \cdot k_{j}+\mu \cdot ks_{j}\right)$, where $k_{i}$ and $ks_{i}$ are the degree and k-shell of node *i*, respectively, and α and μ are adjustable parameters in the range [0, 1]. The node influence is calculated as $wksd_{i}=\sum_{j\in N_{i}}w_{ij}$. It incorporates the node’s k-shell when defining the potential edge weight. However, this method relies on empirical parameters for weight allocation in higher-order neighborhoods, resulting in poor adaptability.

**Measure 5. Equalizing weighted k-shell degree neighborhood (EWKSD).**

EWKSD [57] is based on node degree and k-shell to define potential edge weights. EWKSD extends WKSD by introducing a balance factor λ to coordinate degree scales. It breaks through the limitations of network structure differences. Its edge weight is $w_{ij}=\left({\mathrm{ks}}_{i}+{\mathrm{ks}}_{j}\right)+\lambda \cdot \left(k_{i}+k_{j}\right)$, where λ is the ratio of the average k-shell to the average degree. The node influence is $wksd_{i}=\sum_{j\in N_{i}}w_{ij}$. This approach adapts well to both sparse social networks and dense biological networks without structural adjustment.

**Measure 6. HIC centrality (HIC).**

Most existing methods identify influential nodes from nodal characteristics alone, treating all edges equally in unweighted networks and failing to capture the multifaceted importance of edges. To address this issue, HIC [55] defines a potential edge weight by integrating the H-index, k-shell iteration factor and clustering coefficient of the connected nodes. The potential edge weight is given by $W_{i,j}=\frac{H(i)\cdot Iter(i)+H(j)\cdot Iter(j)}{1+C(i)+C(j)}$, where H(·), Iter(·) and C(·) represent the H-index, k-shell iteration factor and clustering coefficient, respectively. The centrality of node *i* is calculated as $\mathrm{HIC}\left(i\right)=\sum_{j\in \Gamma_{i}}W_{i,j}$, with $\Gamma_{i}$ denoting the neighbor set of node *i*.

**Measure 7. Locality-based structure system (LSS).**

Global centrality measures incur prohibitive computational costs, whereas purely local metrics often overlook critical structural connectivity. To bridge this gap, LSS [56] introduces a parameter-free heuristic that quantifies spreader importance from local topological information. Specifically, the initial weight of node *i* is defined as $w\left(v_{i}\right)=d\left(v_{i}\right)\times ks\left(v_{i}\right)$, where $d(v_{i})$ and $ks(v_{i})$ denote its degree and k-shell value, respectively. For an edge (i,j), the connectivity factor is $cf\left(v_{i,j}\right)=\left(ks\left(v_{i}\right)-\alpha \right)\times \left(ks\left(v_{j}\right)-\alpha \right)$, where α is the number of triangles containing that edge. The total edge weight is then $tw\left(v_{i,j}\right)=\frac{cf(v_{i,j})}{\alpha +1}\times \left(w\left(v_{i}\right)\cdot w\left(v_{j}\right)\right)$. The overall influence of node *i* is given by $\mathrm{LSS}\left(v_{i}\right)=\frac{ks(v_{i})}{n}\sum_{j\in \eta (i)}tw\left(v_{i,j}\right)$, where η(i) is the neighbor set and *n* is the total number of nodes. By fusing degree, positional and clustering information, LSS achieves high accuracy in identifying influential spreaders at a low computational complexity of $O(n{\langle k\rangle }^{2}+|E|)$.

**Measure 8. Hierarchical Neighboring PageRank (HNPR).**

HNPR [54] defines HNPRF as the product of a node’s ks value and the sum of PageRank values of its first-order neighbors. The final score is obtained by multiplying the node’s own HNPRF with its neighbors’ HNPRF. This method integrates global core layer attributes and path propagation characteristics but fails to resolve PageRank’s poor adaptability in undirected networks, and multi-layer multiplication tends to cause excessive score fluctuations.

**Measure 9. Weighted Degree and K-Shell (WDKS).**

WDKS [49] define intrinsic influence as the product of a node’s own degree and ks value, and quantifies neighborhood contributions through potential edge weights. The final node importance is evaluated by the weighted sum of these two components. WDKS is the first method to combine local “quantity” and global “core layer” attributes, but its edge weight calculation does not consider neighbor quality characteristics (e.g., H-index), resulting in insufficient characterization of local connection “quality”.

Despite advancements, hybrid centrality approaches face persistent challenges that remain unresolved in recent studies:

1. **Insufficient index synergy.** Methods such as WDKS [49], HIC [55], and EWKSD [57] integrate local and global attributes through simple weighting, product, or superposition. These linear or isolated multiplicative operations fail to characterize the intrinsic three-way synergy among “quantity” (degree), “quality” (H-index), and “core-layer position” (k-shell).
2. **Crude neighbor contribution quantification.** WDKS reuses node self-attributes (degree × k-shell) as intrinsic influence, while HNPR [54] aggregates neighbors’ PageRank scores without edge-strength regulation. These designs overlook the regulatory role of edge connection strength in influence transmission and suffer from excessive self-attribute interference.
3. **Complexity or parameter dependency.** LSS [56] requires triangle enumeration, limiting scalability. EWKSD relies on an empirically set λ. HNPR inherits PageRank’s iterative cost. These trade-offs prevent seamless application to large-scale networks without structural adjustment.

To address the above issues, this paper proposes an evaluation method based on multi-attribute neighbor contributions (WKDH), which realizes accurate and efficient evaluation through a multiplicative synergy model, an edge-strength-regulated neighbor contribution mechanism, and a topology-driven adaptive weighting framework, all within linear O(m) complexity. Moreover, it does not require parameter adjustment across different network structures, thus exhibiting excellent universality and cross-network stability.

## 3. Proposed Method

This section formalizes the proposed WKDH method to test the following research hypothesis: *If a node’s influence is modeled by the multiplicative synergy of its local connection quantity (degree), local connection quality (H-index), and global core-layer position (k-shell), and if neighbor contributions are regulated by edge-specific strength rather than by the node’s own attributes alone, then such a fusion mechanism can achieve significantly higher consistency with real propagation dynamics than existing hybrid centrality methods, while maintaining linear computational complexity O(m) and remaining free of parameter tuning.*

### 3.1. Rationality Analysis of Multi-Attribute Fusion

The essence of node influence is the comprehensive embodiment of the structural role and functional value carried by nodes in the network topology. It depends not only on the “quality” and “quantity” of local connections but also on the structural position in the global network. A single indicator can usually only describe one aspect of influence, while the organic integration of degree, H-index, and k-shell can more comprehensively capture the true influence of nodes through the synergistic effect of complementary features.

Degree reflects the number of directly connected neighbors of a node, which is an intuitive embodiment of the “quantity” of local connections; H-index characterizes the “quality” of local connections (i.e., the influence level of neighbors) through the degree distribution characteristics of neighbors; k-shell reveals the core layer position of nodes in the global network by iteratively stripping peripheral nodes, reflecting their structural importance. These three indicators describe node characteristics from three dimensions: “local quantity”, “local quality”, and “global position”. The shortcomings of a single indicator can be compensated by the strengths of other indicators.

Moreover, degree, H-index, and k-shell are all indicators with low computational complexity. They do not rely on global path search or iterative convergence, making them suitable for large-scale network scenarios. Their physical meanings are clear and have been widely verified in complex network analysis, ensuring the interpretability and practicality of the fusion model.

### 3.2. Formula Explanation

(1) $${\mathrm{KDH}}_{i}=\lambda \cdot d\left(i\right)\cdot \mathrm{hi}\left(i\right)+\left(1-\lambda \right)\cdot \mathrm{ks}\left(i\right)$$

(2) $$\lambda =\frac{\bar{\mathrm{ks}}}{\bar{d}+\bar{\mathrm{hi}}+\bar{\mathrm{ks}}}$$

(3) $$1-\lambda =\frac{\bar{d}+\bar{\mathrm{hi}}}{\bar{d}+\bar{\mathrm{hi}}+\bar{\mathrm{ks}}}$$

where: d(i) represents the degree of node *i* hi(i) represents the H-index of node *i* ks(i) represents the k-shell value of node *i* λ is calculated based on network topological structure: $\bar{d}=\frac{1}{N}\sum_{i=1}^{N}d\left(i\right)$ (average degree of the network) $\bar{\mathrm{hi}}=\frac{1}{N}\sum_{i=1}^{N}\mathrm{hi}\left(i\right)$ (average H-index of the network) $\bar{\mathrm{ks}}=\frac{1}{N}\sum_{i=1}^{N}\mathrm{ks}\left(i\right)$ (average k-shell value of the network). Through this fusion, we can simultaneously consider the local connectivity, specific attributes, and global structural characteristics of nodes, enabling a more comprehensive evaluation of node importance.

The weighting coefficient λ is adaptively determined by the network’s global topological statistics via Formula (2), eliminating manual tuning. As $\bar{\mathrm{ks}}$ increases, λ enlarges to up-weight the local synergy term d·hi, compensating for the diminished discriminative power of ks when the core-layer structure is pronounced; conversely, a small $\bar{\mathrm{ks}}$ reduces λ to amplify the scarce yet critical global position information. This topology-driven mechanism ensures an automatic balance between local and global attributes across diverse network structures.

(4) $$w_{i,j}={\mathrm{KDH}}_{i}\cdot {\mathrm{KDH}}_{j}$$

This formula is used to calculate the weight $w_{i,j}$ of edge i,j in the network. Here, ${\mathrm{KDH}}_{i}$ and ${\mathrm{KDH}}_{j}$ are the comprehensive feature indicators of node *i* and node *j* obtained based on Formula (1), respectively. By multiplying the KDH values of the two nodes, the comprehensive features at the node level are extended to the edge level, to measure the importance of the edge after fusing multiple characteristics of nodes, and realize the associated mapping from node features to edge features.

(5) $$\bar{w}=\frac{1}{\left|E\right|}\sum_{(i,j)\in E}w_{i,j}$$

This formula is used to calculate the average value $\bar{w}$ of all edge weights in the network. |E| represents the total number of edges in the network. By summing the weights $w_{i,j}$ of all edges in the edge set *E* and then dividing by the total number of edges |E|, we obtain the average level of network edge weights. It serves as a reference value for normalization operations in subsequent calculations, eliminating the influence of factors such as different network scales on edge weight values and providing a comparable basis for relevant calculations of different networks or different nodes in the same network.

(6) $${\mathrm{WKDH}}_{i}=\sum_{j\in N(i)}\frac{{\mathrm{KDH}}_{j}\cdot w_{i,j}}{\bar{w}}$$

This formula is used to calculate the final comprehensive importance index ${\mathrm{WKDH}}_{i}$ of node *i*. N(i) represents the set of neighbor nodes of node *i*. For each neighbor node *j* of node *i*, first calculate the product of the KDH value of the neighbor node and the corresponding edge weight $w_{i,j}$, then divide by the average edge weight $\bar{w}$ for normalization, and finally sum up the calculation results of all neighbor nodes. This not only considers the comprehensive characteristics of the neighbor nodes of node *i* and the weights of the connected edges but also eliminates the influence of the overall edge weight level of the network through the average edge weight, comprehensively measures the importance of node *i* in the network, and integrates the local neighbor relationships and the global edge weight characteristics of the network. The detailed procedure of the WKDH method is summarized in Algorithm 1.
**Algorithm 1** The WKDH Algorithm.**Input:** Complex network G(V,E), where n=|V| (number of nodes) and m=|E| (number of edges)**Output:** Node influence values computed by the WKDH algorithm  1:**for** each node *i* in *V* **do**  2:      Calculate d(i)                   //Compute the degree of node *i*  3:      Calculate hi(i)                  //Compute the H-index of node *i*  4:      Calculate ks(i)                  //Compute the k-shell value of node *i*  5:**end for**  6:**for** each node *i* in *V* **do**  7:      KDH(i)←0                       //Compute KDH by fusing d(i), hi(i), and ks(i) according to Equation (1)  8:      KDH(i)=λ·d(i)·hi(i)+(1−λ)·ks(i)  9:**end for**10:**for** each edge (i,j) in *E* **do**     //Compute edge weight $w_{i,j}$ according to Equation (4)11:      $w_{i,j}={\mathrm{KDH}}_{i}\cdot {\mathrm{KDH}}_{j}$12:**end for**13:sum_w←014:**for** each edge (i,j) in *E* **do**15:      $\mathrm{sum}\_w+=w_{i,j}$16:**end for**17:$\bar{w}=\mathrm{sum}\_w/m$18:**for** each node *i* in *V* **do**19:      WKDH(i)←020:      **for** each node *j* in G.neighbors(i,1) **do**21:            ${\mathrm{WKDH}}_{i}+={\mathrm{KDH}}_{j}\cdot w_{i,j}/\bar{w}$                    //Compute the final WKDH influence value22:      **end for**23:**end for**

## 4. Data Analysis

### 4.1. Datasets

To thoroughly verify the effectiveness of WKDH in evaluating node influence, we use 9 real-world networks (Power [60], Dolphin [61], Jazz [62], Netscience [63], Euroroad [2], Plant [64], Citeseer [65], Faa [66], Infectious [67]) and 6 artificial networks (ER_500, ER_1000, ER_2000, LFR_500, LFR_1000, LFR_2000) for indicator comparison and analysis. Considering the significant heterogeneity of these networks in terms of type, size, and topological structure, selecting them for simulation analysis allows for a comprehensive comparison of the robustness and universality of different methods. These networks are undirected and unweighted.

The network characteristic data are presented in Table 1. Network characteristic indicators depict the network structure from multiple dimensions: |V| represents the total number of nodes, indicating the network scale; |E| represents the number of edges, reflecting the total relationship quantity of node connections. 〈k〉 represents the average degree; $k_{\max}$ represents the maximum degree. 〈d〉 represents the average shortest path length; $d_{\max}$ represents the network diameter; if the network is disconnected, the maximum connected component shall be calculated. 〈c〉 represents the network clustering coefficient. $\beta_{\mathrm{th}}$ represents the infection threshold, a critical value for determining whether an infection can continue to spread.

### 4.2. Evaluation Criteria

**Figure 1:**
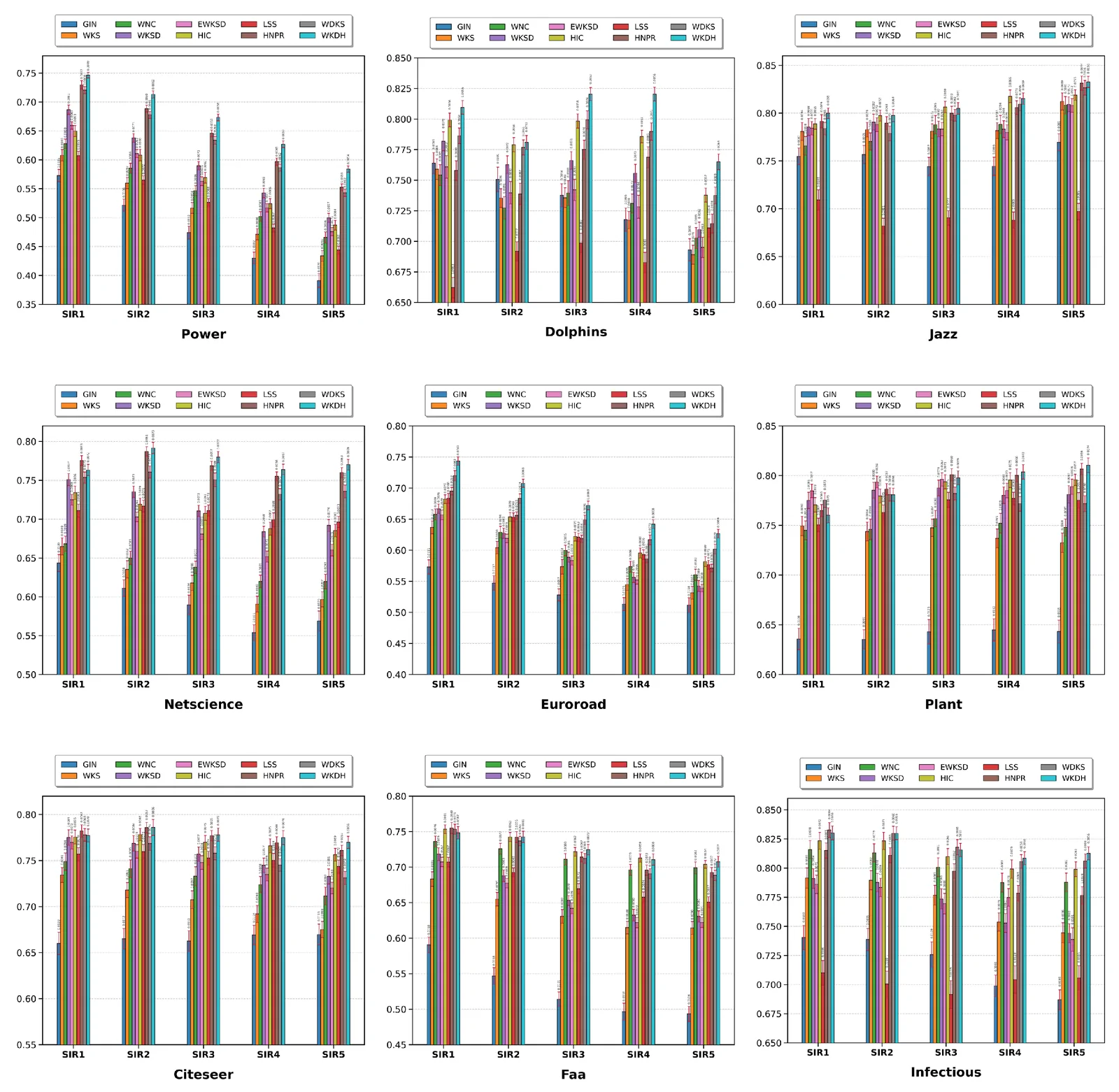
Results of Kendall coefficient calculation for 10 methods in 9 real-world networks. Error bars indicate standard deviations over 100 independent SIR simulations.

**Figure 2:**
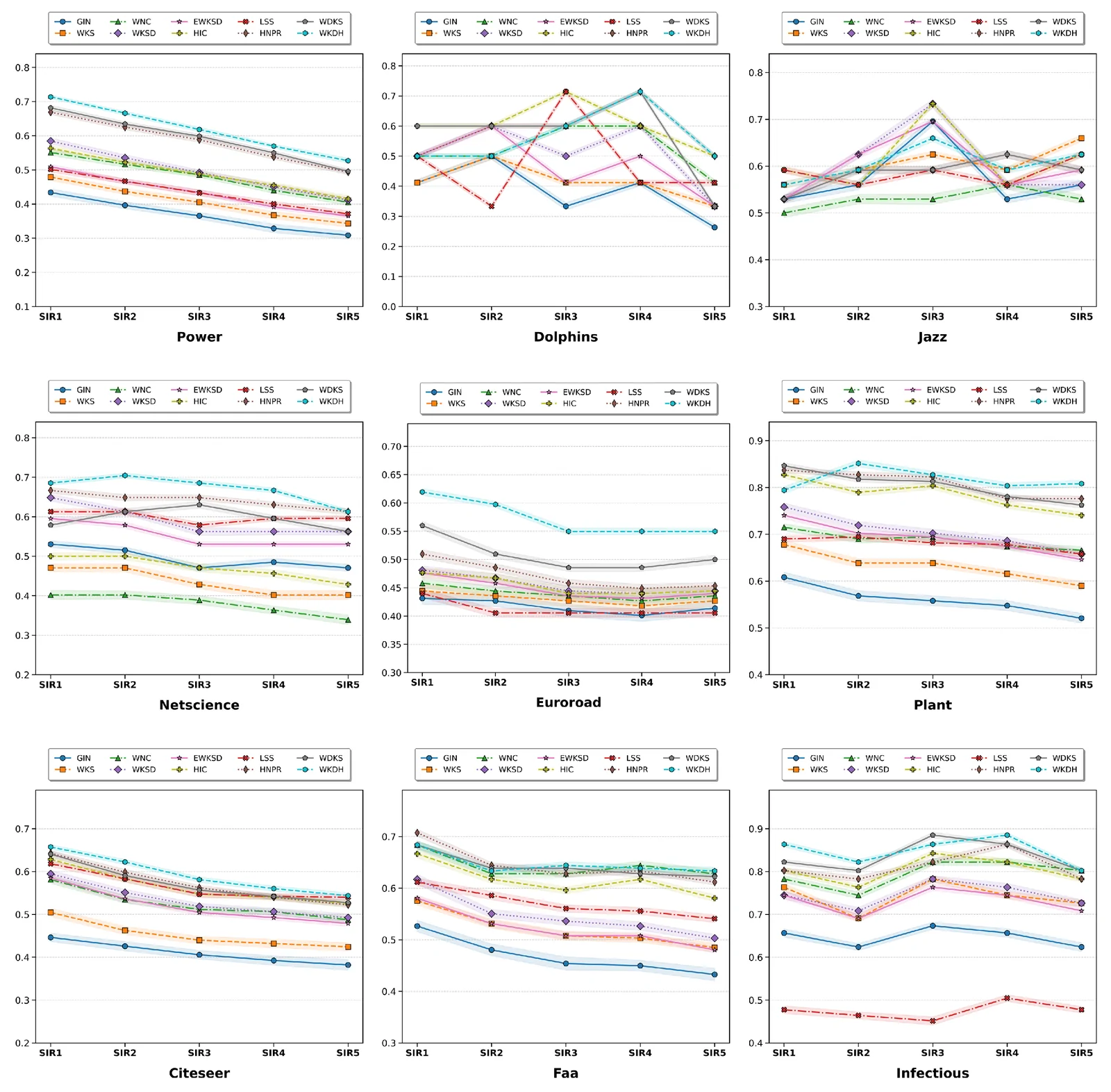
Results of Jaccard coefficient calculation for 10 methods in 9 real-world networks. Shaded regions indicate standard deviations over 100 independent SIR simulations.

**Figure 4:**
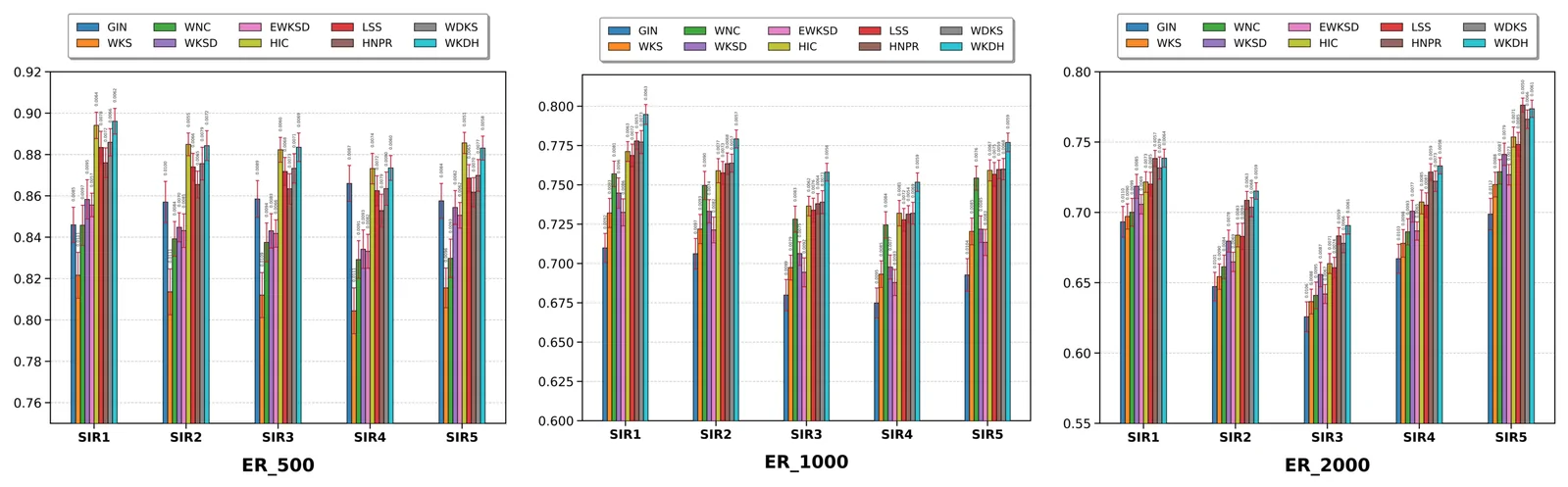
Results of Kendall coefficient calculation for 10 methods in 3 different ER networks. Error bars indicate standard deviations over 100 independent SIR simulations.

The core manifestation of node influence lies in its diffusion ability in information propagation. This paper adopts the classic SIR model [68,69] to simulate the propagation process. The diffusion ability of a node is measured as the proportion of immune and final infected nodes relative to the total number of nodes after the propagation process. In the model parameter setting, the infection rate $\beta_{th}$ is calculated based on the network topological features [55], providing the most appropriate infection probability (threshold) for each network. For the SIR model, five infection probability scenarios ($0.8\beta_{th}$, $0.9\beta_{th}$, $1.0\beta_{th}$, $1.1\beta_{th}$, $1.2\beta_{th}$) are set, and indicator tests are carried out under each scenario to comprehensively reflect the method’s performance under different propagation intensities. The recovery rate γ is fixed at 1. Each result is averaged over 100 independent simulations, with error bars (Figure 1, Figure 4 and Figure 5) and shaded regions (Figure 2) indicating the corresponding standard deviations. The observed variability is consistently smaller than the typical inter-method performance gaps, confirming high stability of the comparative results.

(7) $$\beta_{th}=\frac{\langle k\rangle }{\langle k^{2}\rangle -\langle k\rangle }$$

Here, $\beta_{th}$ denotes the theoretical infection threshold calculated by the SIR propagation model based on network topology, used to define the critical infection probability for information or disease propagation. 〈k〉 is the average degree of network nodes, reflecting the average connection level of nodes, while $\langle k^{2}\rangle$ is the average value of the square of node degrees, reflecting the unevenness of the network degree distribution. These three act together to determine the critical conditions for propagation by leveraging network topological features, assisting the SIR model in reasonably setting the infection probability to make the propagation simulation conform to the actual propagation influence laws of the network.

#### 4.2.1. Kendall Correlation Coefficient

To evaluate the consistency between the node influence ranking results produced by the WKDH method and the true node propagation ability rankings determined by the SIR propagation model, we use the Kendall coefficient [70] as the core quantification indicator.

(8) $$\tau =\frac{2(n_{c}-n_{d})}{n(n-1)}$$

Let τ denote the Kendall coefficient, *n* the total number of network nodes, $n_{c}$ the number of node pairs with consistent relative order in the two rankings, and $n_{d}$ the number of node pairs with inconsistent relative order. The value range of τ is [−1,1]. A value closer to 1 indicates higher ranking consistency. The average value can be calculated under 5 infection probability scenarios to comprehensively evaluate the ranking accuracy of WKDH.

#### 4.2.2. Jaccard Coefficient

In complex networks, node influence typically follows a “head concentration” pattern, where a small fraction of top-ranked nodes plays a dominant role in information diffusion. Therefore, the Jaccard coefficient [47] is introduced for quantitative analysis. To accurately evaluate the matching degree between the WKDH method and the SIR propagation model in identifying high-influence nodes, the top 20% nodes in the network are selected as the core research objects.

(9) $$Jaccard\left(X,Y\right)=\frac{\left|X\cap Y\right|}{\left|X\cup Y\right|}$$

Let *X* denote the top 20% nodes identified by a given method and *Y* the corresponding set of the top 20% nodes identified by the SIR propagation model. |X∩Y| is the number of overlapping elements of the two sets, and |X∪Y| is the number of elements in the union of the two sets. The value range of the coefficient is [0, 1]. A larger value indicates a higher consistency in identifying high-influence nodes, and the performance of the method can be comprehensively tested across five infection probability scenarios.

#### 4.2.3. Imprecision Function

The imprecision function [71] is an indicator used to evaluate the difference in propagation ability between nodes identified by a specific indicator and those identified by the SIR model.

(10) $$\epsilon \left(p\right)=1-\frac{SI(p)}{SI_{\mathrm{eff}}\left(p\right)}$$

where SI(p) represents the total propagation ability of the top *p* nodes identified by a specific indicator, and $SI_{\mathrm{eff}}\left(p\right)$ represents the total propagation ability of the top *p* nodes identified by the SIR model. The smaller the value of the imprecision function, the closer the identification result of the indicator is to that of the SIR model, that is, the better the performance of the indicator in evaluating the node propagation ability.

### 4.3. Benchmark Methods

In this study, the proposed WKDH method is compared with nine established node influence evaluation methods: GIN [58], WKS [19], WNC [59], WKSD [20], EWKSD [57], HIC [55], LSS [56], HNPR [54], and WDKS [49]. These methods encompass both single-index extensions (WKS, WNC) and hybrid centrality approaches that integrate multiple topological attributes (GIN, WKSD, EWKSD, HIC, LSS, HNPR, WDKS). All methods are simulated and implemented under identical experimental settings to ensure a fair and robust performance comparison.

### 4.4. Results and Analysis

In this study, under five SIR infection scenarios of 9 real-world networks, the Kendall coefficient, Jaccard coefficient, and imprecision function are used to comprehensively compare the performance of the WKDH method with the benchmark methods. Meanwhile, artificial networks are introduced, and the Kendall coefficient is calculated to verify its robustness. The effectiveness and stability of the WKDH method in predicting the propagation influence of network nodes are evaluated in a multi-dimensional and systematic manner, providing a solid basis for the practical application value of this method.

#### 4.4.1. Real-World Networks

The Kendall coefficient results in Figure 1 show that WKDH maintains a relatively high consistency with the SIR model’s propagation ability ranking in all five infection scenarios across all datasets, significantly outperforming most baseline methods. In the Power, Jazz, and Euroroad networks, its τ values are higher than those of other methods in different infection scenarios. For example, in the Euroroad network, WKDH achieves higher Kendall coefficients than most baseline methods across infection scenarios, and also outperforms recent hybrid approaches such as HNPR and WDKS. Similarly, in the Dolphins, Netscience, Citeseer, and Faa networks, WKDH maintains a clear leading position in most cases, demonstrating superior ranking ability. In the remaining networks, although the accuracy rate of WKDH is lower than that of the latest methods in some scenarios, the accuracy rate remains relatively high, verifying the excellent performance of WKDH in identifying influential nodes across diverse network structures.

Figure 2 highlights the advantage of WKDH in identifying high-influence nodes. Across five SIR infection scenarios, the matching degree between the actual identification results of WKDH and the SIR model is significantly better than that of most benchmark methods. In the four networks of Power, Netscience, Euroroad, and Citeseer, the Jaccard coefficient of WKDH is higher than that of the compared baseline methods in all scenarios. For example, in the Power network, as the infection rate increases, the Jaccard coefficients of all methods generally show a downward trend. However, the Jaccard coefficient of WKDH always remains at a relatively higher level. In the Plant, Faa, and Infectious networks, WKDH also leads most comparison methods in the majority of cases. For example, in the Plant network, the Jaccard coefficient of WKDH is only lower than that of the HNPR and WDKS methods under the SIR1 scenario, and it is superior to the comparison methods in all other scenarios. These results fully verify the excellent performance of WKDH in identifying high-influence nodes under different network structures and infection scenarios.

**Figure 3:**
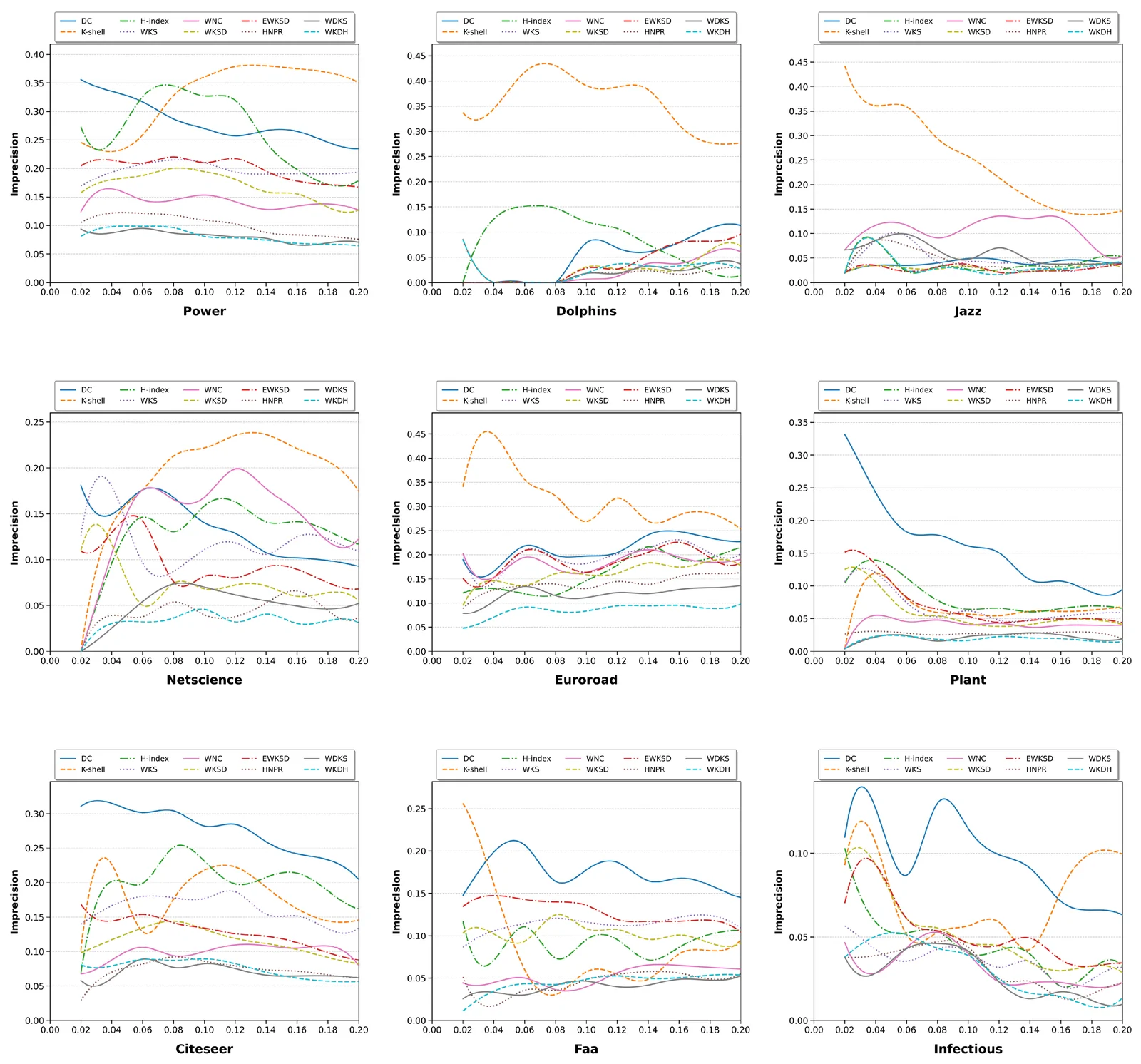
Results of imprecision function calculation for 10 methods in 9 real-world networks.

The high-influence nodes selected through reasonable measurement should demonstrate strong propagation ability. To further evaluate the performance of WKDH, we compare the influence imprecision of high-influence nodes identified by different measurements. The calculation is based on Formula (8), where a smaller ϵ value represents better measurement performance. In the study, the proportion range of high-ranking nodes is set as p∈[0.02,0.2]). As can be seen from Figure 3, the imprecision of WKDH in the four networks of Power, Netscience, Euroroad, and Plant is significantly lower than that of other methods. In the Citeseer network, when p∈[0.15,0.2]), the performance of WKDH is continuously the best. When p∈[0.02,0.15]), although it is not the optimal, the difference from the optimal value is less than 0.02. In networks such as Faa, the difference from the optimal value is also less than 0.01, which fully reflects the excellent performance of WKDH in identifying high-influence nodes and correlating with propagation ability.

#### 4.4.2. Artificial Networks

##### ER Network

The ER network belongs to the category of random graphs. The node degree distribution follows the Poisson distribution, where the degrees of most nodes are close to the average degree of the network, and there are relatively few nodes with extremely large or small degrees. Its construction depends on two parameters: the number of nodes and the generation probability of edges. In this study, ER networks with 500, 1000, and 2000 nodes were generated, and the generation probability of edges was all 0.002. The correctness evaluation is shown in Figure 4.

Figure 4 shows that under ER networks of different scales and SIR infection scenarios, WKDH consistently outperforms benchmark methods in most scenarios. For example, in the ER 500 network, under various SIR scenarios, the Kendall coefficient of WKDH is significantly higher than that of other methods; in the ER 1000 and ER 2000 networks, and similarly, in a large number of SIR infection scenarios, the Kendall coefficient of WKDH is better, indicating that its ranking of identifying high-influence nodes in the ER network has stronger consistency with the actual propagation performance.

##### LFR Network

**Figure 5:**
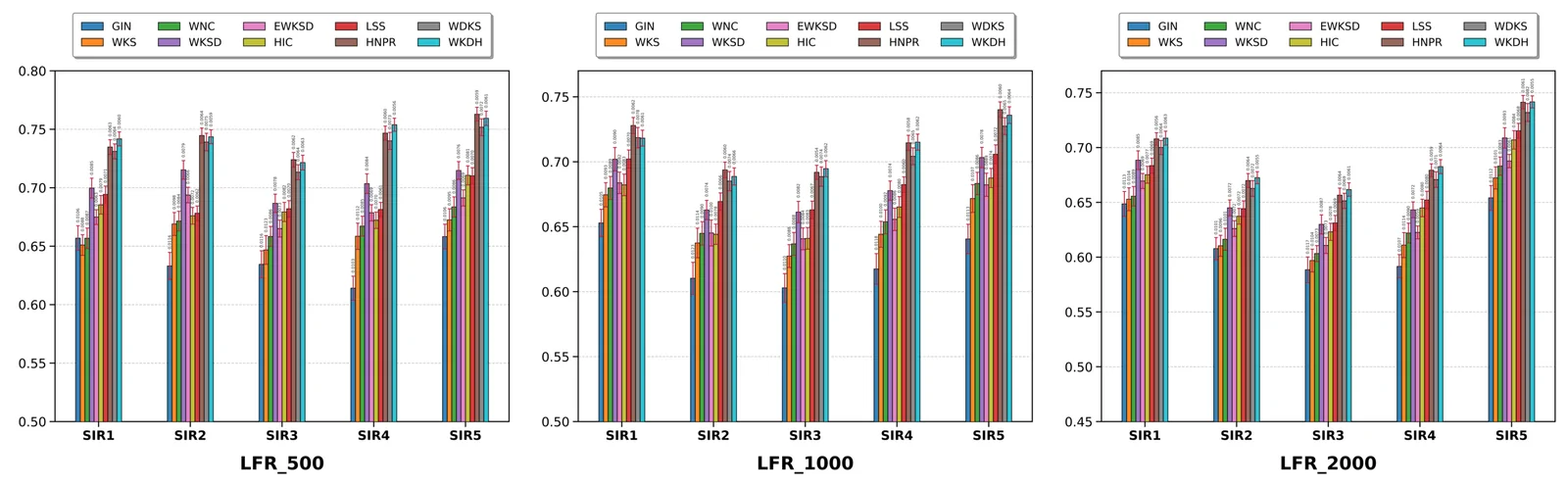
Results of Kendall coefficient calculation for 10 methods in 3 different LFR networks. Error bars indicate standard deviations over 100 independent SIR simulations.

The LFR network is a type of artificial network. It is characterized by following a power-law distribution in terms of degree or community size, producing hub nodes. Key construction parameters include the number of nodes, the power-law exponent of the degree distribution, the power-law exponent of the community size distribution, the minimum number of nodes in each community, the average degree, and the ratio of inter-community edges connected to each node. In this study, LFR networks with 500, 1000, and 2000 nodes were generated. The power-law exponent of the degree distribution was set to 2.5, the power-law exponent of the community size distribution was set to 1.2, and each community had a minimum of 50 nodes. The correctness values of each benchmark method in the LFR network can be found in Figure 5.

From Figure 5, it can be intuitively seen that in various LFR network scales and infection scenarios, the Kendall coefficient performance of WKDH is outstanding. For example, in the LFR 500 network, the Kendall coefficient of WKDH is higher than that of the comparison methods under most SIR scenarios. In the LFR 2000 network, a higher Kendall coefficient is also demonstrated in many cases. In the LFR 1000 network, although it is lower than the HNPR method, when compared with other methods, the Kendall coefficient still maintains a leading position. These results indicate that in the LFR network, the ranking of high-influence nodes identified by WKDH has stronger consistency with the actual propagation situation.

#### 4.4.3. Statistical Significance Analysis

Because many of the observed performance differences among methods are relatively small, relying solely on mean values may lead to unreliable conclusions. To rigorously validate whether WKDH’s superiority is statistically significant, we conducted a non-parametric Friedman test followed by a Nemenyi post hoc analysis across the 9 real-world networks. Each combination of network and infection rate was treated as an independent test instance, yielding 45 blocks (9 networks × 5 SIR scenarios) for the Kendall correlation coefficient.

The Friedman test rejected the null hypothesis of equal performance among the 10 methods ($\chi^{2}\left(9\right)=316.8$, p<0.001), confirming significant differences. The average ranks in Table 2 show that WKDH achieved the best overall performance (avg. rank = 1.33), followed by HNPR (3.00), WDKS (3.22), and HIC (3.33).

The Nemenyi post-hoc test (α=0.05, critical difference = 2.02) further reveals that WKDH is statistically superior to WKSD, WNC, EWKSD, LSS, WKS, and GIN. While no significant difference is detected between WKDH and HNPR, WDKS, or HIC, WKDH achieves the best average rank (1.33) among all competing methods.

We note that the Friedman–Nemenyi test is applied only to Kendall’s τ because it directly evaluates full-ranking consistency, the primary criterion for centrality comparison. The Jaccard coefficient and imprecision function rely on fixed or varying proportion thresholds (top-20% and p∈[0.02,0.2]), whose absolute values are sensitive to network size and violate the exchangeability assumption required by the Friedman test. Extending the same statistical framework to these metrics remains for future work.

### 4.5. Parameter Sensitivity Analysis

**Figure 6:**
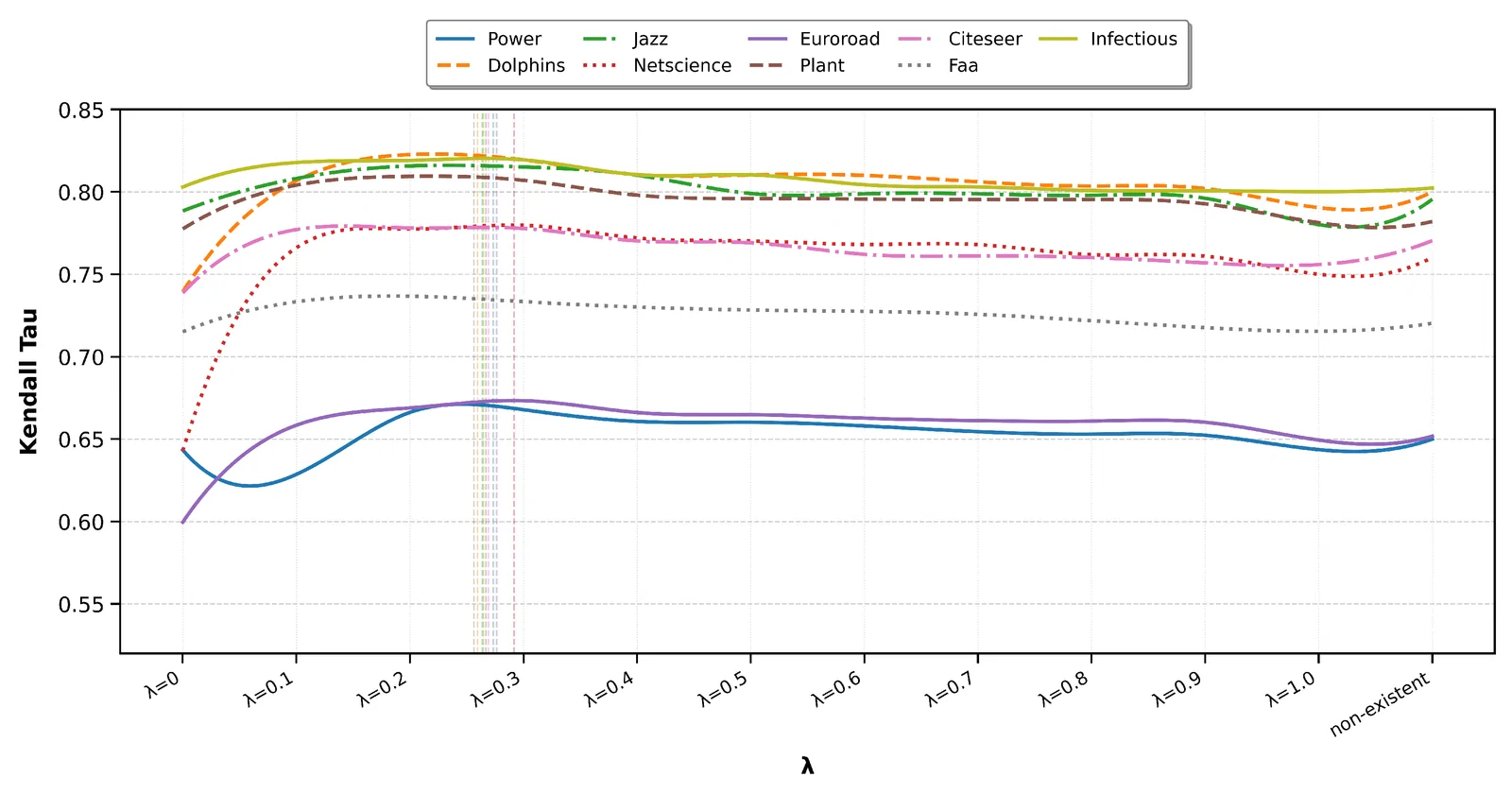
Parameter sensitivity analysis of WKDH on 9 real-world networks.

To validate the adaptive design of λ, we vary it from 0 to 1.0 (step 0.1) across the 9 real-world networks and include a “non-existent” case where the weighting mechanism is removed. As shown in Figure 6, the adaptive λ values from Formula (2) (vertical dashed lines) consistently lie within or near the peak regions of the Kendall coefficient curves, and the flat profiles around these maxima indicate low sensitivity to perturbations. Notably, extreme values (λ=0 or λ=1) and the removal of λ both yield substantial performance drops, confirming that neither pure global positioning (ks) nor pure local synergy (d·hi) suffices. An adaptive balance between local and global attributes is indispensable, and the topology-driven λ achieves this automatically.

### 4.6. Network Dismantling Analysis

To evaluate the effectiveness of WKDH in identifying nodes whose removal dismantles the network, we conduct a targeted node-removal experiment on the 9 real-world networks. Specifically, nodes are removed sequentially in descending order of their importance scores computed by each method, and the network connectivity degradation is recorded after each removal. The degradation is quantified as the relative size of the largest connected component with respect to the original network. An effective influence identification method should cause rapid connectivity degradation when its top-ranked nodes are removed, indicating that the identified nodes are truly critical to maintaining network structure.

**Figure 7:**
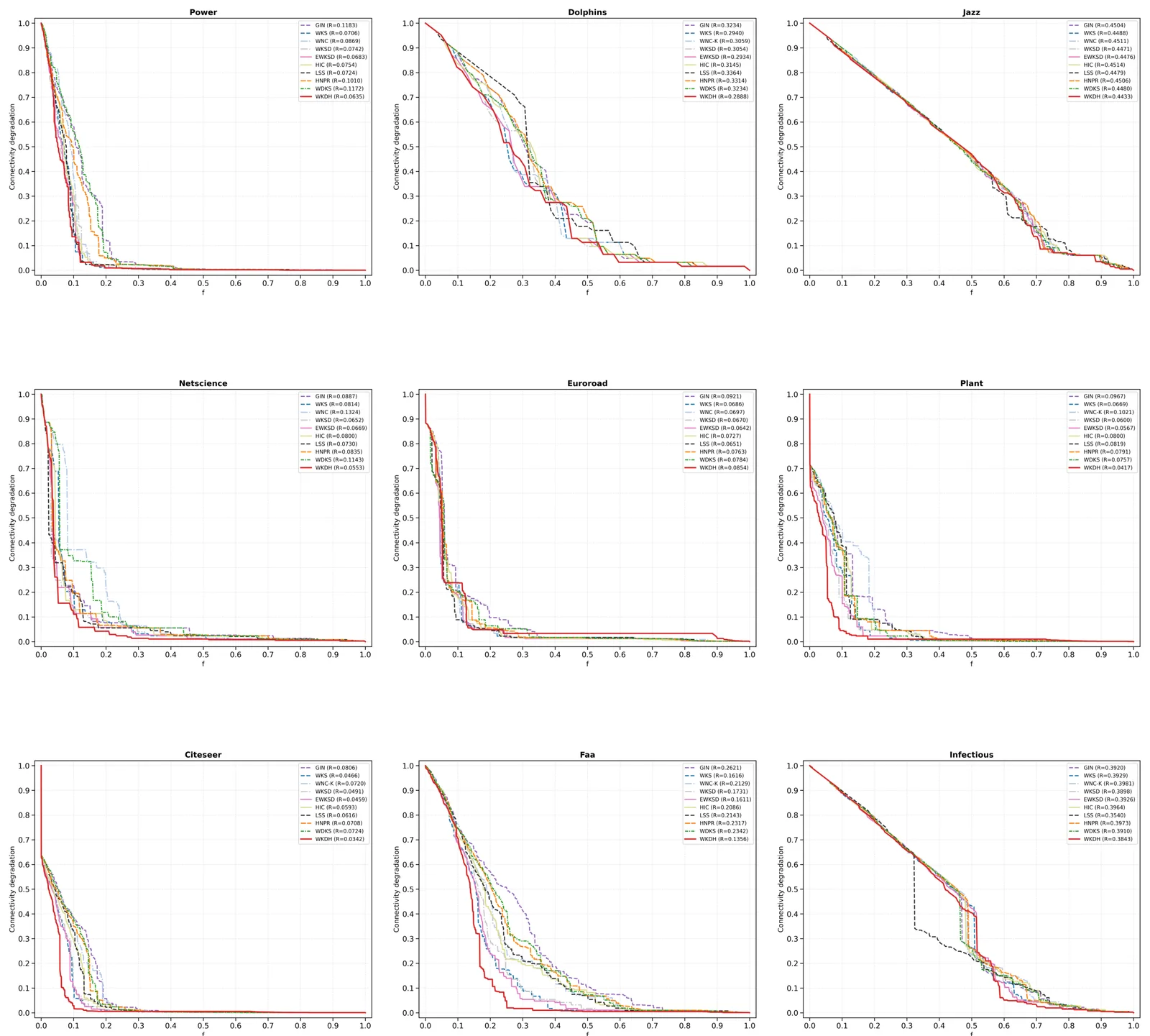
Network Dismantling Analysis on 9 real-world networks via sequential node removal.

Figure 7 illustrates the connectivity degradation curves for all 10 methods across the 9 networks. The robustness metric *R* (area under the degradation curve) is reported in the legend for each method; a smaller *R* signifies better identification of structurally vital nodes. As shown in the figure, WKDH achieves the smallest *R* in 7 out of 9 networks (Power, Netscience, Jazz, Euroroad, Plant, Citeseer, and Faa) and remains highly competitive in the remaining networks (Dolphins and Infectious). For instance, in the Plant network, WKDH (R=0.0417) substantially outperforms the second-best method, indicating that the nodes identified by WKDH are most destructive to network connectivity upon removal. In dense networks such as Jazz and Infectious, where the curves of all methods are relatively close, WKDH still maintains performance comparable to the best baselines. These results demonstrate that WKDH is highly effective in dismantling diverse network topologies and densities, and that its ranking of influential nodes reliably reflects the actual structural importance of nodes under targeted removal.

## 5. Conclusions

This paper proposes WKDH, a node influence evaluation method based on the contribution of multi-attribute neighbors, designed to accurately identify key influence nodes in complex networks. WKDH innovatively integrates both the local structural attributes and global structural attributes of nodes. By constructing a weighted evaluation model, it deeply excavates the synergistic effects among different attributes, breaking through the limitations of simple weighting or superposition in traditional methods. This method has a linear computational complexity of O(m) and is suitable for large-scale network scenarios. Moreover, WKDH does not require parameter adjustment according to different network structures and has excellent stability and universality.

The experimental results show that on 9 real-world and 6 artificial networks, WKDH performs excellently in influence ranking, high-influence node identification, and propagation, outperforming nine benchmark methods under diverse SIR infection scenarios. The WKDH method effectively balances identification accuracy and computational efficiency, providing a reliable tool for structural analysis of complex networks, optimization of information propagation, and enhancement of network robustness. It has important theoretical value and practical application significance.

While the experimental results demonstrate the effectiveness and robustness of WKDH, several limitations should be acknowledged. First, the evaluation is conducted on a finite collection of 9 real-world and 6 synthetic networks; although these networks span diverse domains and topological characteristics, they do not exhaust all possible network structures. Second, the current study focuses exclusively on static, undirected, and unweighted networks. The behavior of WKDH in dynamic networks with temporal evolution, in directed networks with asymmetric influence propagation, or in weighted networks where edge intensities carry intrinsic significance, remains to be investigated. Third, while Section 4.6 evaluates the effectiveness of WKDH in network dismantling via targeted node removal, its robustness against random edge removal, network noise, or incomplete data has not yet been systematically examined. Fourth, the formal statistical validation is currently limited to Kendall’s τ; analogous non-parametric tests for the Jaccard coefficient and imprecision function are deferred to future work due to threshold-dependent heterogeneity across networks. These limitations delineate the boundary of the current work and motivate the following directions for future research.

Future research can further explore its incremental update mechanism in dynamic networks. By tracking the dynamic changes of nodes and edges to evaluate node influence in real time, it can adapt to the dynamic characteristics of actual networks. In addition, refining edge weight definitions for weighted and directed networks can better optimize performance. In weighted networks, integrating the original weight of edges with node attributes and in directed networks, distinguishing between in-degree and out-degree attributes to design differentiated contribution degree calculation rules, can further improve the adaptability across diverse network types.

## Figures and Tables

**Table 1:** Various characteristic indicators of 9 real-world and 6 artificial networks.

Network	|V|	|E|	〈k〉	$k_{\max}$	〈d〉	$d_{\max}$	〈c〉	$\beta_{\mathrm{th}}$
Power	4941	6594	2.6691	19	18.9892	46	0.0801	0.3483
Dolphins	62	159	5.129	12	3.357	8	0.259	0.1723
Jazz	198	2742	27.697	100	2.235	21	0.6180	0.0266
Netscience	379	914	4.8232	34	6.0419	17	0.7412	0.1424
Euroroad	1174	1417	2.414	10	18.3951	62	0.0167	0.5002
Plant	1745	3098	3.5507	71	8.2944	26	0.1447	0.0997
Citeseer	3265	4536	2.7786	99	9.3105	28	0.1446	0.1688
Faa	1226	2410	3.9315	34	5.929	17	0.0675	0.1567
Infectious	410	2765	13.4878	50	3.6309	9	0.4558	0.0564
ER_500	500	258	1.032	5	12.2857	30	0.0	0.8897
ER_1000	1000	1008	2.016	9	8.9469	25	0.0022	0.4912
ER_2000	2000	4045	4.045	12	5.5725	13	0.0024	0.25
LFR_500	500	1036	4.144	12	4.5484	8	0.0065	0.2454
LFR_1000	1000	2091	4.182	14	5.0009	10	0.0059	0.2404
LFR_2000	2000	4229	4.229	13	5.4367	10	0.0027	0.238

**Table 2:** Average ranks of the 10 methods based on Kendall’s τ across 45 test instances (9 networks × 5 SIR scenarios).

Rank	Method	Avg. Rank	vs. WKDH
1	WKDH	1.33	—
2	HNPR	3.00	∘
3	WDKS	3.22	∘
4	HIC	3.33	∘
5	WKSD	5.67	•
6	WNC	6.44	•
7	EWKSD	6.67	•
8	LSS	7.22	•
9	WKS	8.56	•
10	GIN	9.56	•

∘ = no significant difference; • = significantly worse than WKDH (Nemenyi, α=0.05, CD=2.02).

## Data Availability

The original contributions presented in this study are included in the article. Further inquiries can be directed to the corresponding author.
